# Barriers to and Facilitators for Using Nutrition Apps: Systematic Review and Conceptual Framework

**DOI:** 10.2196/20037

**Published:** 2021-06-19

**Authors:** Laura Maria König, Christiane Attig, Thomas Franke, Britta Renner

**Affiliations:** 1 Department of Psychology University of Konstanz Konstanz Germany; 2 Faculty of Life Sciences University of Bayreuth Kulmbach Germany; 3 Department of Psychology Chemnitz University of Technology Chemnitz Germany; 4 Institute for Multimedia and Interactive Systems University of Lübeck Lübeck Germany

**Keywords:** nutrition apps, mHealth, digital health, usage facilitators, usage barriers

## Abstract

**Background:**

Nutrition apps are effective in changing eating behavior and diet-related health risk factors. However, while they may curb growing overweight and obesity rates, widespread adoption is yet to be achieved. Hence, profound knowledge regarding factors motivating and hindering (long-term) nutrition app use is crucial for developing design guidelines aimed at supporting uptake and prolonged use of nutrition apps.

**Objective:**

In this systematic review, we synthesized the literature on barriers to and facilitators for nutrition app use across disciplines including empirical qualitative and quantitative studies with current users, ex-users, and nonusers of nutrition apps.

**Methods:**

A systematic literature search including 6 databases (PubMed, Web of Science, PsychINFO, PSYNDEX, PsycArticles, and SPORTDiscus) as well as backward and forward citation search was conducted. Search strategy, inclusion and exclusion criteria, and the planned data extraction process were preregistered. All empirical qualitative and quantitative studies published in German or English were eligible for inclusion if they examined adolescents (aged 13-18) or adults who were either current users, ex-users, and nonusers of nutrition apps. Based on qualitative content analysis, extracted individual barriers and facilitators were grouped into categories.

**Results:**

A total of 28 publications were identified as eligible. A framework with a 3-level hierarchy was designed which grouped 328 individual barriers and facilitators into 23 subcategories, 12 categories, and 4 clusters that focus on either the individual user (goal setting and goal striving, motivation, routines, lack of awareness of knowledge), different aspects of the app and the smartphone (features, usability of the app or food database, technical issues, data security, accuracy/trustworthiness, costs), positive and negative outcomes of nutrition app use, or interactions between the user and their social environment.

**Conclusions:**

The resulting conceptual framework underlines a pronounced diversity of reasons for (not) using nutrition apps, indicating that there is no “one-size-fits-all” approach for uptake and prolonged use of nutrition apps. Hence, tailoring nutrition apps to needs of specific user groups seems promising for increasing engagement.

## Introduction

Overweight is one of today’s most urgent public health issues [1,2]. It is related to a number of noncommunicable diseases including cardiovascular diseases, cancer, diabetes, premature deaths, and reduced quality of life [3-5]. Furthermore, it poses substantial economic costs on society [6]. Currently, more than 2 billion people worldwide are affected and figures are projected to rise further [2,7]. Thus, interventions are urgently needed to curb growing overweight and obesity rates by promoting weight loss, weight maintenance, or preventing weight gain [2]. Ideally, these interventions are not only effective, but also affordable and reach a large number of people around the globe.

The widespread adoption of mobile phones [8] thus constitutes a promising opportunity to reduce overweight. Accordingly, using mobile phones and other mobile devices for health purposes (mobile health or mHealth; [9,10]) has become increasingly common [11], and the majority of mHealth apps available in app stores [12] target weight-related behaviors such as eating behavior [13,14]. Moreover, mobile app–based interventions have been shown to be effective in improving diet and diet-related health outcomes [15] and effect sizes of these interventions are comparable to those of traditional nondigital interventions [15]. However, a large share of the general population does not yet use nutrition apps [16-18]. To promote their use and thus to put their full potential into effect, further knowledge about reasons for and against adoption and prolonged use of nutrition apps is necessary.

These reasons are likely multifaceted. For instance, research in the area of wearable health devices (eg, fitness trackers, smartwatches) has already shown a large variety of reasons for device uptake, sustained use, and abandonment related to, for example, health status, health-related goals, (de)motivation, perceived utility, measurement inaccuracy, usability, convenience/accessibility, and privacy [19,20]. However, these findings may not be easily transferrable to nutrition apps as they differ in central aspects. For instance, nutrition apps usually require manual initiation of data recording by opening the app or pressing buttons, and often also manual input of consumed foods to provide feedback, for example, by having users enter foods and estimated serving sizes using a comprehensive food database [21]. Fitness apps and wearables, by contrast, allow for automatic data collection based on various sensor technologies (eg, accelerometers or gyroscopes). Hence, the reliability of these 2 mHealth services refers to different data sources—the user versus technology—and might thus be perceived differently by users. Furthermore, the difference in active versus passive data collection might impact the motivation to use the services long term, as tracking food intake manually might require more effort, when compared with tracking physical activity automatically [17]. Finally, mHealth services for nutrition and physical activity differ in the type and temporal pattern of feedback provided. Because of automatic and continuous collection of (in)activity data, wearable fitness trackers can provide feedback continuously, even while an activity is taking place, which allows for immediate adjustments. For nutrition apps, by contrast, data (and thus feedback), are provided for distinct eating occasions. Moreover, feedback is often only available after the food was consumed or at least chosen. Thus, immediate adaptation of the behavior might not be possible, which might impact how feedback is perceived and evaluated. Therefore, barriers and facilitators for nutrition apps may differ at least in part from barriers and facilitators for the use of fitness apps and wearables. Finally, these differences might also explain why fitness apps are generally more popular than nutrition apps [17].

Two systematic reviews have identified several factors influencing engagement with mobile weight loss and weight maintenance interventions. These factors include personalization, simplicity, entertainment, usability, social support, and the presence of certain features such as self-monitoring, prompts, and feedback [22,23]. However, nutrition apps can be used for a variety of goals, including self-monitoring [24], eating healthier [25], or even gaining weight [26], which again may reflect a variety of underlying motivations including health status (*cf*. [19]) and specific needs and expectations. Furthermore, the methodological focus of the reviews was restricted. For example, Lyzwinski et al [23] included only qualitative studies. While qualitative research allows for a great richness of participants’ responses, quantitative research usually comprises larger samples and may allow for formal testing of theory. Most studies included in Sharpe et al [22], by contrast, were randomized controlled trials which either tested the effect of the presence or absence of certain features (eg, social network, tailored content) on engagement indices or evaluated features of a specific intervention, which might not be generalizable to all mobile eating interventions. It was thus deemed necessary to review the literature more broadly to reflect a wider range of app-based nutrition interventions, study designs, and user perspectives. Accordingly, this present systematic review aimed to synthesize the literature on barriers to and facilitators for the uptake, continued use of, and disengagement from nutrition apps in the general population. This was done to provide a comprehensive overview of factors that hinder or promote use to be utilized as starting points for nutrition app development and optimization. It includes studies with all possible user groups (nonusers, users, ex-users, and not specified) [17] as well as both qualitative and quantitative studies on a wide range of available nutrition apps.

## Methods

### Protocol and Review Design

A protocol was prepared and published on the Open Science Framework [27] prior to completion of data extraction. This review reports on the generation of an overview of the evidence. The second goal (designing a questionnaire based on the results) will be presented elsewhere. Reporting is guided by the PRISMA guidelines [28] (Multimedia Appendix 1).

### Data Sources and Search

We searched the following databases before May 2019: PubMed, Web of Science, PsychINFO, PSYNDEX, PsycArticles, and SPORTDiscus. For PubMed, Web PsychINFO, PSYNDEX, PsycArticles, and SPORTDiscus. A Boolean search term was used for this purpose: ((“nutrition app*” OR “diet app*” OR “weight loss app*” OR “weight control app*” OR “weight management app*” OR “food journal” OR “health app*” OR “personal quantification” OR “quantified self” OR “personal informatics”)) AND (adoption OR adherence OR abandonment OR attrition OR barriers OR motivation OR attitude OR *engagement OR “former user” OR ex-user OR “*continued use”). However, a slightly modified term was used for Web of Science due to differences in use of the asterisk (ie, we had to add “disengagement” and “discontinued use”). No restriction was placed on publication date. Moreover, we conducted backward citation search by manually screening the reference lists of included studies for additional relevant references. We also conducted a forward citation search in Google Scholar using the included studies to complement the data search.

### Study Selection

#### Inclusion and Exclusion Criteria

Eligible studies examined factors hindering uptake or continued use (ie, barriers) or factors promoting update or continued use of nutrition apps (ie, facilitators). To be eligible for inclusion, studies had to examine adolescents (aged 13-18) or adults in the following participant groups: current users, ex-users, or nonusers of nutrition apps either for themselves or for their children. Studies with children, adolescents younger than 13, and health care providers using nutrition apps for patient support were excluded. Only empirical articles were included (ie, literature reviews, meta-analyses, and conference abstracts were excluded). All study designs including qualitative or quantitative methodologies were eligible for inclusion. Studies had to investigate general nutrition app use (ie, studies evaluating particular apps, for instance, in intervention settings, were excluded). Moreover, studies focusing more broadly on the use of health apps were included as long as they specifically stated that nutrition apps were included. Studies were only eligible if the examined apps included an assessment of diet, such as logging consumed foods (eg, studies evaluating sole weight logging apps were excluded). Further, we included only English and German articles. Finally, 2 studies known to authors from other sources were also included [29,30].

#### Screening

Authors LK and CA independently reviewed titles and abstracts, and, subsequently, full texts according to the inclusion and exclusion criteria. Conflicts were resolved through discussion until consensus was achieved.

### Data Extraction and Collation

Authors LK and CA reviewed each full-text article independently and extracted data on facilitators and barriers to nutrition app use (ie, direct quotes from qualitative studies, questionnaire item texts from quantitative studies). Subsequently, all authors categorized facilitators and barriers manually according to principles of qualitative content analysis (ie, inductive category development) [31]. Differences in abstraction were resolved by discussion until consensus regarding category logic (ie, no overlaps of contents across categories) was achieved. The inducted categories were first defined, then compared and harmonized with the extracted quotes. The final category system including the underlying quotes was documented in an MS Excel spreadsheet and can be found in Multimedia Appendices 2 and 3. Categories may contain both barriers and facilitators as several aspects of nutrition apps and their use were not universally perceived to be positive or negative (eg, app usability can both be a facilitator and barrier depending on the individual user’s perception). In a final step, the categories were grouped into higher-order clusters based on their origin or source as they might provide further insight into starting points for improvement.

In addition, author LK reviewed each full-text article and extracted the following study characteristics into MS Excel spreadsheets: app user group (all, users, or users and ex-users), sample size, age, gender, specificities of the study sample (general population or patients), study design (qualitative, quantitative, mixed), study location, and type of app (nutrition vs health app; see Multimedia Appendix 4).

## Results

### Literature Search

**Figure 1:**
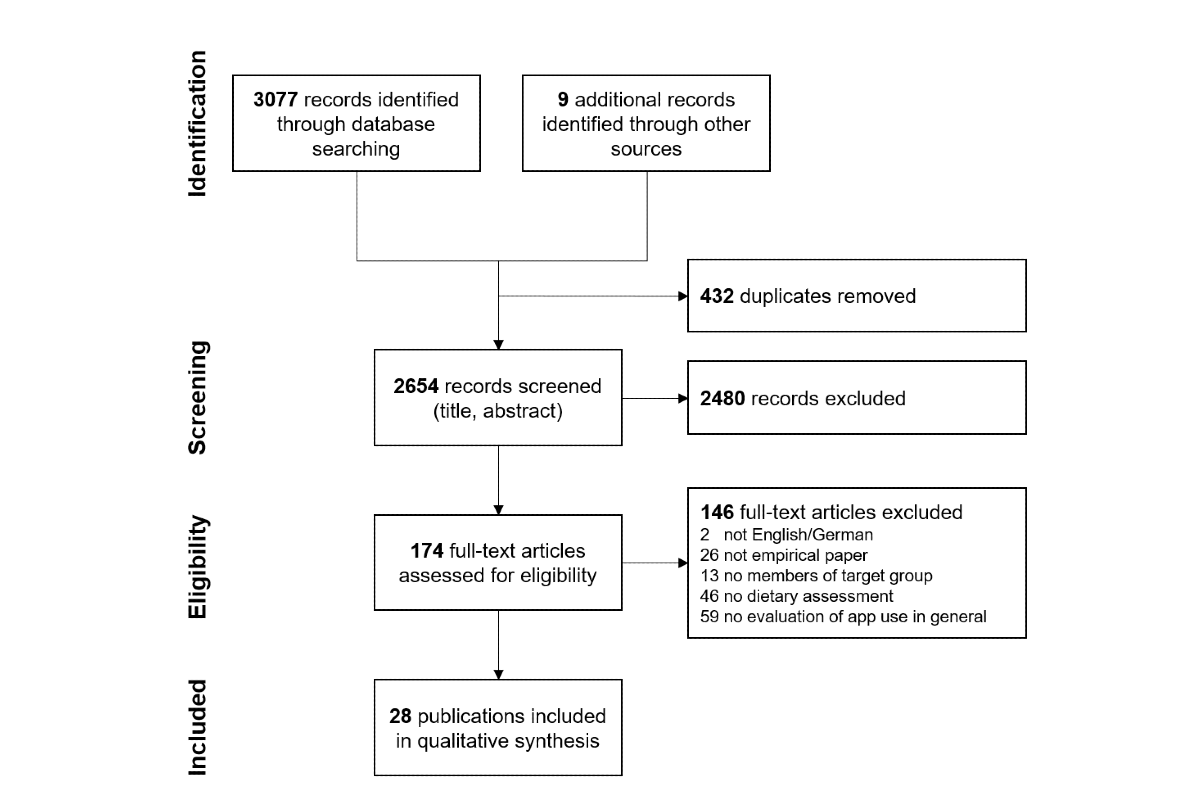
Flow diagram of article selection.

A total of 2654 individual records were screened. After 2480 were excluded by screening titles and abstracts, 174 full texts were screened for eligibility. Subsequently, 28 publications containing 30 studies [24-26,29,30,32-54] were included (see Figure 1 for a flow diagram). Of these, 9 publications were identified through other sources: 7 were identified through forward and backward citation search [25,26,35,39,41,45,47], and 2 were known to the authors through unrelated literature searches [29,30].

### Characteristics of the Included Studies

The 28 publications were all published between 2012 and 2019. Two publications contained 2 studies [49,53], while the remaining 26 publications each reported results from 1 study [24-26,29,30,32-48,50-52,54]. Almost all samples comprised adults from the general population, while 1 publication [45] focused on adolescents and 1 publication [26] focused on women with an eating disorder. Eight publications [26,33,34,36-38,44,45] focused specifically on barriers and facilitators of nutrition app use (eg, diet tracking features in MyFitnessPal, LoseIt!), while 20 publications [24,25,29,30,32,35,39-43,45-48,50-54] investigated barriers to health apps in general, but explicitly included nutrition apps such as calorie counters. Fifteen of 28 [24,25,29,30,32,38,41,43,45-47,51-54] included publications did not focus on a specific user subgroup, while 5 publications [35,36,39,48,50] focused on users and 8 [26,33,34,37,40,42,44,49] included both users and ex-users. For the majority of publications, data were collected in the United States (15/28) [24-26,29,30,32,34,35,40,41, 43,45,51,52,54], followed by the UK (3/28; [37,38,47]) and Canada (2/28; [33,48]). One publication presented data from multiple countries. Ten of the 28 publications referred substantially to theoretical models for the deduction of hypotheses or discussion of results, while the remaining studies were not theory driven. See Multimedia Appendix 4 for an overview of included studies.

### Barriers to and Facilitators for Nutrition App Use

A total of 328 barriers to and facilitators for nutrition app (non)use were extracted from the publications. The number of extracted barriers and facilitators varied greatly between publications (mean 11.71 [SD 8.75]; range 2-39).

**Figure 2:**
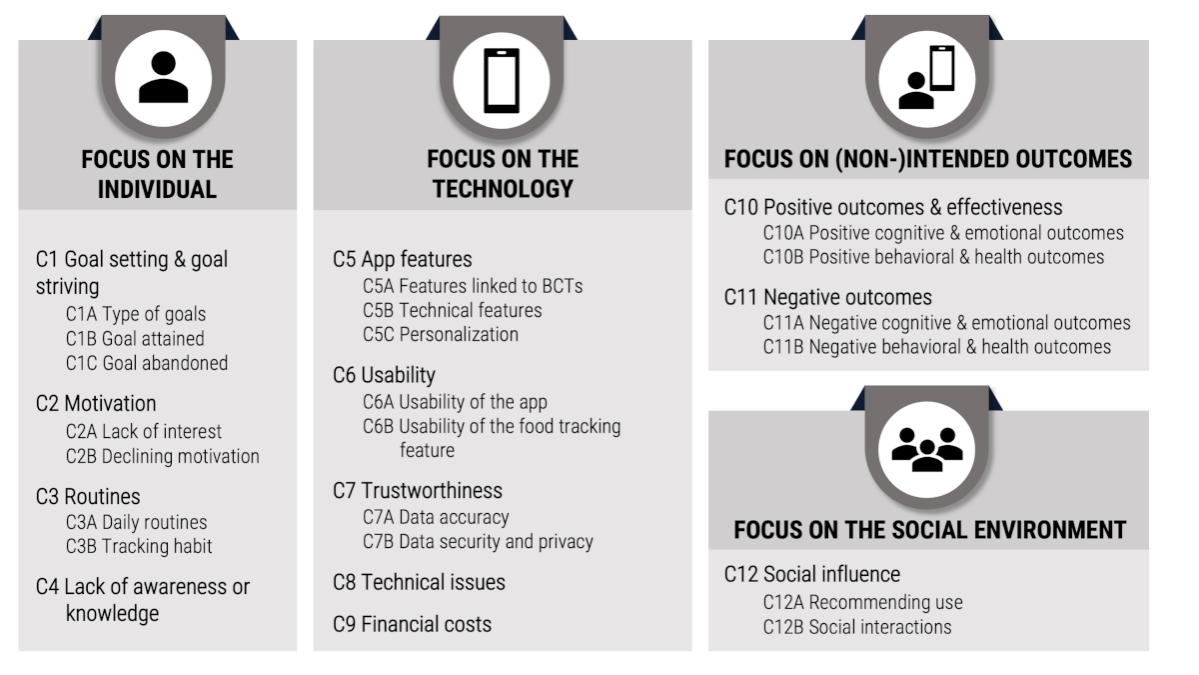
Framework comprising 12 categories C1-C12 and respective subcategories of barriers and facilitators identified in the individual studies. BCTs: behavior change techniques.

While grouping barriers and facilitators into categories, a 3-level hierarchical framework emerged (see Figure 2 for a schematic overview of the framework). First, barriers and facilitators were grouped into 23 subcategories. Second, several categories were clustered (eg, lack of interest and declining motivation were both related to motivation, thus grouped together), resulting in 12 categories (C1–C12; note that C4, C8, and C9 do not contain subcategories). Third, the resulting categories were grouped into higher-order clusters that focus on either (1) the individual (C1–C4), (2) the app and the smartphone (C5–C9), (3) intended and nonintended outcomes of nutrition app use (C10 and C11), or (4) social influence (C12).

In the majority of study results (23/30), barriers and facilitators were not distinguished by user group (ie, no differentiation regarding barriers or facilitators for uptake, use, or long-term use). Therefore, such a differentiation is also not reflected in the presented framework. However, a differentiation regarding study samples was possible and can be inspected visually in Multimedia Appendix 2.

Barriers and facilitators within the individual were related to C1 goal setting and goal striving (ie, C1A the type of goal; C1B goal attained; C1C goal abandoned), C2 motivation (ie, C2A lack of interest; C2B declining motivation), C3 tracking routines (ie, C3A daily routines; C3B tracking habit), and C4 lack of awareness or knowledge. Barriers and facilitators related to the app and the smartphone were C5 app features (ie, C5A features linked to behavior change techniques [BCTs]; C5B technical features; C5C personalization), C6 usability of the app (C6A) and the food tracking feature (C6B), C7 trustworthiness regarding data accuracy (C7A) as well as data security and privacy (C7B), C8 technical issues, and C9 financial costs of the app. In addition, barriers and facilitators were identified that stem from using the app, which include intended and nonintended positive outcomes and effectiveness (ie, C10A positive cognitive and emotional outcomes; C10B positive behavioral and health outcomes) as well as C11 negative outcomes (ie, C11A negative cognitive and emotional outcomes; C11B negative behavioral and health outcomes). Finally, barriers and facilitators stemming from C12 social influence (ie, C12A recommendation to use; C12B social interactions) were identified.

There was a substantial variation in the number of subcategories targeted by the different publications (mean 6.79 [SD 3.87]; range 1-16 of 23 subcategories) and accordingly, the frequency of the subcategories being mentioned across publications varied substantially (mean 8.26 [SD 4.21]; range 1-19 of 28 publications; see Multimedia Appendix 2 for details).

### C1 Goal Setting and Goal Striving

#### C1A Types of Goals

In 15 publications [24-26,29,30,32,33,35-42], the type of goal was mentioned as a facilitator for nutrition app use. A variety of goals were identified ranging from highly specific nutrition-related goals to very general health-related goals, or even to improvements in other aspects of life. The majority of goals were related to nutrition, for example, food tracking [24,26,32,33], diet improvement [25,34-36], and weight management [32] including both weight loss [26,29,35,37,38] and weight gain [26]. In addition, changing other health behaviors was mentioned [30,33,35,39-41], for example, physical activity [35] as well as adopting a new or maintaining an existing behavior [35]. Further participants named more general goals such as improving their health [42], to be more mindful or to find balance, needing assistance with medical or health-related decision making, increasing their knowledge in order to answer specific questions, finding triggers, being able to ask their physician more specific questions or to ask for a second opinion, curing or managing a condition, or executing a treatment plan [39,41]. Finally, some participants identified further goals related to other aspects of life and new life experiences, such as maximizing work performance [39]. These results highlight that nutrition apps may be used for a great variety of different goals; apps may thus need to be explicit about which goals they target to attract appropriate users and so to facilitate uptake and long-term use.

#### C1B Goal Attained; C1C Goal Abandoned

Two reasons for disengagement with an app which were related to goals were identified. On the one hand, app use may no longer be necessary if the goal was reached or a desired habit was formed [29,33,43,44], which was identified in 4 publications. On the other hand, 1 publication highlighted that goals may be abandoned and the app along with it [29].

### C2 Motivation

#### C2A Lack of Interest

In 4 publications [24,30,43,45], nonusers expressed a general lack of interest in using health apps including nutrition apps because they felt that they did not need to use one [24,30], for example, because of other available tools such as paper and pencil diaries [43], or competing interests such as preferring to use their smartphones for other apps (eg, social media) [45].

#### C2B Declining Motivation

Furthermore, in 7 publications [24,33,36,44,46-48], some (ex)users reported that their motivation to use apps could decline over time [24,36,44,46], for example, because of limited progression [33] or boredom [6], for instance, because the app only provided a limited range of functionalities [48].

### C3 Routines

#### C3A Daily Routines

Four of the reviewed publications [29,32,33,47] highlighted that the fit between the user’s daily routines or current living situation and app might impact uptake and adherence [33]. For instance, participants in 1 study [29] discontinued using an app because they were not able to use it in certain environments (eg, at work). Others criticized that using an app interfered with their daily activities and social life [32]. Dennison et al [47] highlighted that apps were most likely to be utilized if they were well integrated into users’ typical smartphone use patterns. Thus, according to the reviewed studies, nutrition apps need to fit to the user’s daily routines, for example, by not being able or not wanting to use the app for inputting data while at work.

#### C3B Tracking Habit

In 4 publications [40,44,47,49], tracking habits (or a lack thereof) was identified as an influencing factor. Some participants stopped using an app because they forgot to use it in daily life [44,47,49]. Similarly, Yuan et al [40] showed that apps were less likely to be abandoned when a habit of using the app was formed. Nutrition apps might therefore need to include features that facilitate establishing a tracking habit to promote their use.

### C4 Lack of Awareness or Knowledge

Four publications [30,33,43,45] highlighted the importance of knowledge and skill especially for nutrition app uptake. Some of the nonusers surveyed in the reviewed studies reported not to be aware that health and nutrition apps existed [30,43]. Others may be aware of this type of apps, but are unsure which one to use, lack awareness of specific functionalities and capabilities, or do not know how to use them properly [33,43,45].

### C5 App Features

#### C5A Features Linked to Behavior Change Techniques

In a total of 14 of the included publications [29,33,36-39,42,43,45-50], the inclusion of features that can be linked to the BCT Taxonomy version 1 [55] was evaluated as a positive factor. Participants explicitly expressed their satisfaction with comprehensive food databases for self-monitoring (BCT category 2) of food intake [37,48] and criticized databases that were too limited [49]. Regarding feedback, the opportunity to view a history of tracked data without visiting a medical doctor or having the opportunity to send data to health professionals remotely [50] was appreciated. Similarly, participants in the study by Aljuraiban [46] were more likely to discontinue use if monitoring by a specialist was not offered. By contrast, there were disagreements on how messages should be designed [33]: some participants stated that they did not like to count calories [38] or to restrain themselves based on feedback from the app [47]. Regarding information presentation, participants suggested using various media formats (eg, video, audio) [45] and visualizations [42]. They were keen on the fact that a high level of detail was preserved in the feedback [47] and preferred to access information on the go [37]. Participants generally valued the provision of nutrition knowledge (BCT category 4) that they did not already have or might not be able to access otherwise [36,38,43]. Finally, rewards (BCT category 10) were appreciated [37,39,42,43,45]; however, gamification elements were seen favorably by some [36,43] and perceived as demotivating by others [36].

#### C5B Technical Features

In 9 studies [33,36,37,42,43,47,48,50,51], participants mentioned the inclusion of further technical features. For instance, participants highlighted the need for integration with other apps, for example, to synchronize calorie consumption and expenditure [33,42]. Poor integration with other apps might lead to disappointment with an app and to subsequent disengagement [33]. Integration of location tracking was criticized as unnecessary in Dennison et al [47]. However, based on the included quotes, one can only speculate whether these features will actually lead to abandonment of an app or simply be ignored. Finally, receiving messages, prompts, and reminders to use the app were not universally appreciated. While some participants stated that they were helpful [36,37,43,47], others reported to be annoyed by too frequent notifications [33,47,48,51], for example, to update the app regularly to ensure its functionality [50].

#### C5C Personalization

Ability to personalize apps was mentioned in 11 publications [29,33,36-38,43,45-48,50]. Features should be customizable and tailored to individual needs and goals [29,36-38,43,46,50]. For instance, participants valued the opportunity to set customized reminders, to have choices in message content and tone [47], and to receive personalized information and coaching through an app [45]. Some participants stated liking apps that provided a prespecified list of goals to choose from [29,33], while others stated that they would prefer to set individual goals as they were unsatisfied with the ones provided [33,36]. In addition, tailoring to ethnic and age-specific (eg, adolescence) preferences was valued [45,48].

Moreover, it might be valuable to tailor the features implemented in an app to the users’ needs. In several studies, the absence of desired or helpful features [33,42,48] was mentioned, which may lead to abandonment of the app [29]. By allowing users to customize the app, personal autonomy, freedom of choice, and a feeling of congruency are preserved, which might prolong use [36]. Thus, as needs and expectations regarding the design and content of certain app features may vary between users, customization or variation of both features and app content in general may thus be helpful to prevent abandonment.

### C6 Usability

#### C6A Usability of the App

In 19 publications [24,25,30,33-39,43,45-50,52,53], usability of the app was seen as an important precursor of nutrition app uptake and continued use. Participants criticized that apps were too confusing, complex, and stressful, both when setting them up (eg, because of lengthy instructions) [35,47] and when using them [24,33,35,38,43,45-49]. Accordingly, easy and simple tracking procedures were valued by users [39] and perceived ease of set up and use increased the likelihood for adoption and continued use [25,30,33,34,36,37,50]. In a similar vein, participants reported disliking apps that were time-consuming to use [36,47,52], which might lead to increased levels of stress [53]. Lack of time might thus be considered an important barrier to using nutrition apps [43]. Finally, interface design aspects were also mentioned in a few studies. Specifically, studies highlighted that an attractive design may increase the likelihood for an app to be used [37,45].

#### C6B Usability of the Food Tracking Feature

Food tracking features are an integral part of nutrition apps. The importance of their usability was highlighted in 7 of the reviewed studies [24,33,39,42,44,48,49]. Especially, the ease of use of food databases was seen as a critical component. Many users of nutrition apps reported to have experienced difficulties when entering data [44,48], for example, regarding the correct identification of foods because of too many options [33], finding the correct foods because they were missing from the database [49], or entering the correct foods and portion sizes [33]. Providing detailed entries and entering homemade food or meals consumed at a restaurant were seen to be especially challenging [49]. Participants might even cease to enter their meals, for instance, when consuming a variety of foods over a longer time span [49]. By contrast, Lieffers et al [33] noted that participants preferred larger over smaller food databases, as these saved time and were convenient.

Furthermore, the time needed for food journaling was seen as crucial by participants [33,42,49]. Entering foods being too time-consuming was named as an important reason for not using or ceasing to use nutrition apps [24,44]. Participants would therefore prefer automated tracking functions [39] or food scanners [37]. However, Lieffers et al [33] noted that several participants preferred using an app-based food database over other nondigital tracking methods because apps were perceived as more convenient and less time-consuming.

### C7 Trustworthiness

#### C7A Data Accuracy

A (lack of) concern regarding data accuracy was identified in 7 publications [33,36,37,42,47-49]. While some participants saw nutrition apps as a trusted ally that supported them in achieving their goals [36], other participants were concerned regarding the accuracy and trustworthiness of information presented in the app [42]. Some participants expressed concern regarding human error when tracking food intake, as tracking tools might allow deliberately adjusting entries, which may lead to inaccurate records [47]. Other participants stated being concerned about tracking errors within the database or the app itself [47,49], for example, missing or duplicate food entries or incorrect caloric information [33], or about being unsure whether the provided information, for example, in discussion boards, was accurate and could be trusted [37,47]. Moreover, participants criticized that apps may be misleading regarding the predicted accuracy of provided information [48].

#### C7B Data Security and Privacy

A number of data security concerns were mentioned by participants in 10 of the reviewed publications [7,24,29,35,36,42,43,47,50,51]. Some participants were unsure what the data might be used for without their awareness [47]. They expressed worry that their potentially sensitive data would be made available to third parties such as health insurers [50] or companies, for example, to tailor advertisements to them [47], although they would not consent to the data being shared as these are private matters and could be exploited [43]. In particular, location detection was seen critical because it might be a risk to personal safety, for example, when GPS data were accessed by burglars [42,47]. Zhou et al [51] specifically focused on data security concerns and barriers and facilitators to the use of mHealth apps including nutrition apps. Concerns were raised because of storing unencrypted personal data on users’ smartphones, sending data to remote servers without permission, and a general lack of privacy statements in many apps. Accordingly, participants were unsure whether they could trust the apps and their developers [24,29], and apps provided by health experts such as medical doctors were seen as more trustworthy and persuasive [47]. In one study, participants expressed that they would trust apps by organizations more than apps by commercial companies, and that they would trust apps that were branded or labeled [43]. Indicators for a credible source may thus be important for nutrition app uptake. Other participants, however, stated to be unconcerned because they thought their data would not be of interest to third parties [47].

Especially in apps with in-app community features or connections to social media, anonymity was important to app users [36]. However, there were different opinions as to whether participants approved of sharing data with a community: Some current users were favorable toward sharing their data online [42], whereas for others, sharing data without one’s awareness was a frequently named reason for abandoning an app [24]. Sharing might be disliked especially if participants feel that they cannot control which and how much information is shared [47]. Besides ensuring that data are stored safely and privacy is respected, it may thus also be important to make data protection efforts transparent to potential users.

### C8 Technical Issues

An important prerequisite for being able to use nutrition apps is compatibility of the app with one’s smartphone [29]. However, even if the smartphone fulfills an app’s basic system requirements, a number of technical issues might decrease the likelihood of app adoption and continued use. Accordingly, technical issues were identified as a potential barrier in 8 publications [24,29,33,42,43,47,48,51]. Apps may slow down the smartphone [51] and thus may impair the use of other apps or phone functions [47] or lead to crashes [33]. Furthermore, excessive battery drain [29,43,47], use of memory or storage [43,47], and use of mobile data [24] were criticized in several studies. Moreover, technical issues within the app such as app dysfunctions [48] and inconvenient data transfer [42] may lead to frustration and subsequent disengagement. Finally, users might disengage from using an app because of concerns related to radiation from their smartphones [43]. App developers therefore might need to find a balance between using advanced but resource-intensive features and ensuring compatibility with many different smartphones that vary in age and technical features.

### C9 Financial Costs

Financial costs of nutrition apps were mentioned in 8 publications [24,30,33,35,40,43,46,51]. While few users may be more motivated to use the app because they paid for it [33], the price and costs of in-app purchases mainly hindered app use [24,30,43], especially because there are free apps available [35,51]. Some participants, however, said that they might be willing to pay some money for the app if it was good and provided a good value for money [35,40]. Furthermore, some participants criticized hidden costs (eg, for enabling additional app features) [46], which may lead to disengagement [24]. Thus, financial costs might need to be low and made transparent to promote nutrition app use.

### C10 Positive Outcomes and Effectiveness

#### C10A Positive Cognitive and Emotional Outcomes

In 13 publications [25,29,32,34-38,40,42,43,50,53], participants reported a number of positive outcomes of nutrition app use that were related to cognitions and emotions. Regarding cognitive outcomes, using an app may increase users’ awareness and motivation for healthy eating [25,34,35,37,43,50,53] and induce positive feelings, including feeling energized and healthy [29,32]. Accordingly, nutrition apps are perceived to be informative and to promote nutrition knowledge [38,53]. Tracking may also provide participants with a sense of accountability and the ability to track progress over time [29] and improve their self-efficacy [36]. Regarding emotional outcomes, participants reported that using an app made them feel good about themselves [53] and their bodies [32]. Furthermore, apps may provide encouragement and support [35,38], and using them can be fun and enjoyable [34,40] which may positively influence engagement [42].

#### C10B Positive Behavioral and Health Outcomes

Positive impact of nutrition app use on behavior and health [25,34], potentially due to improved self-management skills [50], was reported in 6 publications [25,30,34,47,50,54]. Accordingly, nutrition apps are seen as potentially useful [34,54], as merely viewing behavioral data may facilitate behavior change [47]. However, not all surveyed participants agreed with this notion and perceived nutrition apps to not be effective in changing health and related behaviors [30,47].

### C11 Negative Outcomes

#### C11A Negative Cognitive and Emotional Outcomes

A number of potential adverse cognitive and emotional consequences of app use were reported in 8 studies [32,33,37,38,42,47-49]. Participants reported obsession with food or calorie counting [32,33,38,49] and being overly engaged with one’s states and behaviors [42]. Moreover, negative app-generated information including feedback based on tracking of food intake and messages sent by the app might evoke negative emotional reactions including disappointment, guilt, and anxiety [32,33,37,48,49], especially if users fall short of reaching a predetermined goal [47]. Finally, apps may also make users feel neurotic about their body image [32].

#### C11B Negative Behavioral and Health Outcomes

Using nutrition apps might also have a negative impact on behavior and subsequently health, which was reported in 7 studies [26,33,43,44,48,49,52]. Some participants expressed worry that eating foods that are unhealthy, but easy to log (eg, ready meals) would be rewarded, which may encourage their consumption [44,49]. Other participants expressed concern that feedback on calorie consumption might backfire, for example, when caloric intake and expenditure are tracked in combination and burned calories may be seen as a permission to eat more [33]. Finally, some participants stated being concerned that apps promoting extreme calorie restriction might even lead to potential harm [48], including inducing or exacerbating an eating disorder [26]. Nutrition app use might therefore be promoted if expected or experienced negative consequences are attenuated.

### C12 Social Influence

#### C12A Recommending Use

Social influence might promote app uptake, as was highlighted in 9 publications [24,29,30,35,43,45,48,51,54]. For instance, participants may learn about apps from family members or friends [29,30,35,43], from a health or fitness professional, or from their employer [24,29]. Still others stated to have chosen the app based on recommendations and positive reviews in app stores, social media, or TV [29,30,54].

#### C12B Social Interactions

Similarly, 11 publications [33,42-45,47-49,51,53,54] indicated that social interactions in the app or related to the app might influence nutrition app use. For instance, participants valued competitions [43,48] and support functions [37]. Furthermore, Wang et al [53] reported that sharing data over the internet might sustain motivation in users. When their friends stopped using the app, participants reported that it was more likely that they would stop using the app as well [49]. However, some participants also reported that learning about others’ success or competing against other app users might be demotivating [33,43,49].

By contrast, perceived undesirability and stigmatization of using apps might hinder uptake and continued use [45,47,49] because it may be embarrassing [47]. Some participants stated that they did not feel comfortable using an app in front of others [33,44] or did not even want other people to know that they were using an app because it might imply that they have a certain disease [51]. Thus, social influence might both promote and hinder nutrition app uptake and use, depending on whether attitudes toward nutrition apps in the social environment are positive or negative.

## Discussion

### Principal Findings

Nutrition apps are less popular than fitness apps [17], although they might have comparably beneficial effects on health [15,56]; for instance, on body weight reduction [15]. Hence, to enable large-scale health effects such as tackling the obesity epidemic [57], acceptance, wide-spread adoption, and long-term use of nutrition apps need to be enhanced. To this end, it is necessary to better understand differences and dynamics in use. This systematic review provides a hierarchical framework of barriers and facilitators for nutrition app use. The framework highlights that besides technological reasons, characteristics of the (potential) user, the interplay between user and technology, and the social environment impact whether a nutrition app is used. For instance, it underlines the importance of tailoring the app content to the user’s goals, expectations, and needs. As Villinger et al [15] pointed out, nutrition apps mainly employ 4 categories of BCTs [55,58] that primarily address constructs of deliberate behavioral control, such as goal setting, self-monitoring, and feedback (see also [59] for an analysis of commercial apps). However, as some (prospective) nutrition app users may decide what or how much to eat based on intuition rather than based on deliberation, the number of nutrition app users might be increased, for instance, by developing apps that address a preference for intuition [17].

The broad range of barriers and facilitators identified in this review may be due the great variety of samples and study designs included that exceeds previous reviews on the topic. For instance, 9 of the 11 studies included in Sharpe et al [22] were evaluations of randomized controlled trials or ongoing weight management programs. Researchers might thus have been especially interested in usability evaluations of specific program features that support long-term engagement with the tested intervention. Consequently, their synthesis puts an even stronger emphasis on barriers and facilitators related to technology. When including surveys of general population samples that are not restricted to participation in an intervention study, more barriers and facilitators may be identified that might not play a role for study participants. For example, lack of awareness or knowledge might not emerge as a barrier when evaluating a digital weight management program because participants often attend a training session before the start of the study (eg, [60]). Similarly, technical issues might not be as frequent, as participants might be preselected based on the type of smartphone they use (eg, [61]), or they might receive a smartphone from the study team to ensure compatibility (eg, [62], Study 3). Besides, data security concerns might not be of great importance when taking part in a study at a university, as researchers might be seen as more trustworthy [47]. Finally, financial costs might not play a role because using an app as part of a study is usually free. Thus, by explicitly including studies independent of the use of specific nutrition apps, this review was able to generate a comprehensive list of barriers and facilitators that play a role when deciding whether to use (or continue to use) a nutrition app in daily life.

Previous reviews also often focused on specific user groups such as current users of nutrition apps [22] or remote tracking technology [19] or on ex-users of wearables [20], and thus lack the perspective of potential users who did not yet think about using mHealth technology for health promotion or decided against its use (for a discussion, see also [17]). This review, by contrast, included literature on nutrition apps in general and included current and ex-users of nutrition apps as well as nonusers of nutrition apps and thus generated an extensive list of barriers and facilitators. Consequently, several subcategories were predominantly or even exclusively identified in publications which included ex-users or nonusers in addition to current nutrition app users. For instance, the category C4 (lack of awareness or knowledge) was not mentioned in publications that exclusively focused on current users, and the subcategory C2A (lack of interest) was only identified in publications which also included nonusers. However, one could argue that these barriers are especially important to address in order to facilitate contemplation of the use of nutrition apps (eg, through medical prescription which is now possible in Germany [63]), which is an important first step in the nutrition app adoption process [17,64]. Similarly, the category C3 (routines) with its subcategories C3A (daily routines) and C3B (tracking habit) was not identified in studies which focus exclusively on current users, presumably because a significant number of current users have already established a tracking habit and are using an app that fits their daily routines. Although certainly not all current users of mHealth technology already have established a tracking habit, those that do are likely to use the technology for periods of a year or more [20,65]. Thus, forming a habit of using a nutrition app might be an important prerequisite for prolonged nutrition app use (see [66] for a discussion of the importance of habits for behavior change). To be able to take habit formation into account when developing nutrition apps, further research is needed to identify app features (eg, reminders, identification of event-based cues [67]) that are most beneficial for establishing a tracking habit.

Despite the differences in target populations and technologies, there is substantial overlap between barriers and facilitators to different mHealth technologies identified in this review and previous reviews [19,20,22,23]. For instance, reviews on both nutrition apps and wearables identified facilitators and barriers related to data security and privacy, app features, or technical issues. Thus, many of the identified barriers and facilitators could be generalized across mHealth technologies, and the presented framework may provide insights for designing mHealth technologies more broadly.

### Implications for the Development of Nutrition Apps

The framework developed in this review summarizes barriers and facilitators for nutrition app use based on empirical investigations and can therefore guide development of theory and measurement instruments, for example, questionnaires to assess interindividual factors related to app uptake. Moreover, the identified barriers and facilitators, specifically on the level of technology (C5–C9) as well as outcomes of user–technology interaction (C10–C11), can inform the improvement of nutrition apps. To this end, 8 design guidelines derived from the presented results are listed in Table 1. First, usability was the most frequently identified barrier in this review and was also identified in previous reviews [19,20,22,23]. Importantly, usability issues were identified both concerning the app in general (design guideline 1; eg, navigation through the app) and more specifically concerning the food tracking feature and the underlying food database (design guideline 2). The latter constitute core features of many nutrition apps [68,69]. Previous research has shown that usability issues related to tracking of food intake might impact willingness to record eating events [70]. Therefore, deficient usability of the food database might also indirectly impact other categories of barriers and facilitators identified in this review such as accuracy and trustworthiness if fewer meals are recorded. Subsequently, this may also impact features that rely on accurate data, such as feedback. Usability of nutrition apps and especially food tracking features should therefore be a major concern for app developers. User burden can, for example, be reduced by using simpler input mechanisms, such as by indicating portion sizes using common household items [71], or by photo-based food recording [68,72].

**Table 1:** Design guidelines based on review results.

Design guideline	Related references
DG1^a^: Enhance app usability through quick set up, avoidance of lengthy instructions, and high ease of use.	[24,33,35,38,43,45-49]
DG2: Enhance usability of the food tracking feature through simple input mechanisms and enabling quick and reliable identification of the correct foods (eg, by including automated tracking functions or barcode scanners, indicating portion sizes, photo-based food recording).	[24,33,37,39,42,44,48,49,68,71,72]
DG3: Include effective behavior change techniques, for example, goal setting, self-monitoring and providing feedback, rewards, and shaping knowledge.	[22,23,36,37,48,73-75]
DG4: Allow for personalization of the app to fit individual needs and goals (eg, give choice to enable or disable gamification elements or reminders, offer customizable reminders, and adaptability to personal variables such as ethnic preferences or age group).	[19,22,23,29,33,36-38,43,45-48,50,59,76]
DG5: Anticipate possible outcomes of nutrition app use to promote positive outcomes (eg, increase of awareness and motivation for healthy eating, improvement of self-management skills, and nutrition knowledge) and avoid negative outcomes (eg, obsessive calorie counting, feelings of guilt, disappointment, or anxiety).	[23,25,29,32,34-38,40,42,43,47,50,53,54,77-79]
DG6: Advance trust in data accuracy by restricting opportunities for human error (eg, when tracking food intake) and enhancing data transparency (eg, specify source of nutritional values).	[33,37,42,47-49]
DG7: Enhance data authority by providing transparency regarding data sharing with companion and third-party apps and giving the choice to prohibit data transfer.	[24,29,42,43,47,48,51]
DG8: Be economical regarding use of smartphone resources (eg, avoid excessive memory and mobile data usage and battery drain).	[24,29,33,43,47,51]

^a^DG: design guideline.

The presence of certain app features including self-monitoring and feedback features can be seen as a facilitator for continued nutrition app use (see also [22]). Specifically, in this review, several app features could be identified that can be subsumed under BCT categories included in the BCT Taxonomy [55], such as self-monitoring (BCTs 2.3 and 2.4), feedback (BCTs 2.2 and 2.7), or goal setting (BCTs 1.1 and 1.3). As Lyzwinski et al [23] pointed out, the inclusion of BCTs is often valued by nutrition app users. It could thus be concluded that the inclusion of certain BCTs (design guideline 3) might not only increase effectiveness of interventions [73,74] but also engagement [75]. Moreover, both this review and previous reviews highlighted that users appreciated opportunities for personalization of the app [19,22,23], which is also related to effectiveness in the literature [59,76]. It can therefore be recommended to include features such as feedback, goal setting, and prompting, and to allow for customization, for example, by allowing users to set customized reminders, to increase engagement (design guideline 4). Many BCTs (eg, 4.3 reattribution, 4.4 behavioral experiments, 5.2 salience of consequences), however, were not mentioned by the study participants. Future research therefore needs to investigate their effects on nutrition app uptake and prolonged use.

Moreover, anticipated or experienced positive and negative outcomes, including the (lack of) effectivity, were among the most frequently identified reasons for nutrition app (non)use. Interestingly, positive and negative outcomes of use were only rarely addressed in previous reviews. For example, potential negative consequences of using nutrition apps such as feelings of guilt were only explicitly addressed in Lyzwinski et al [23]. From a psychological point of view, however, anticipated or perceived positive and negative consequences for one’s health are important precursors of engaging in a behavior (eg, [77,78]). Proximal outcomes, which include cognitive and emotional consequences of use, might be especially important for continuously performing a behavior [77]. Accordingly, outcome expectancies are central components of models of (health) behavior including Social Cognitive Theory [80] and the Health Action Process Approach [81]. It could therefore be recommended to anticipate potential negative outcomes of using an app to prevent them. At the same time, potential positive consequences of nutrition app use, including positive emotional consequences (eg, increased well-being [79]) should be emphasized more to promote use (design guideline 5).

Another critical factor influencing the acceptance of nutrition apps is data accuracy. In the area of nutrition apps, this factor is closely related to the data quality of the underlying data base, which opens up room for human error by allowing users to add their own entries. Consequently, incorrect nutritional values and uncertainty regarding their source might follow [33,37,42,47-49]. If human error cannot be completely avoided, opportunities should be created to increase transparency regarding data sources and thus trust in the system (design guideline 6). Furthermore, barriers in the area of privacy protection were identified [24,29]. In order to strengthen the data sovereignty of users, concerns regarding the transfer of data to third parties should be addressed by making them transparent and optional (design guideline 7). Finally, aspects of sustainability and energy efficiency play a role in the acceptability of nutrition apps [29,43,47]. Manufacturers should thus take care to design their apps sparingly in terms of data storage and energy use (design guideline 8).

Previous reviews have already highlighted a lack of theory when developing the content of app-based interventions (eg, [56,82]). However, when aiming to understand factors related to engagement with nutrition apps, less than half of the publications included in this review used theories such as the technology acceptance model [83] or the theory of planned behavior [84] to design the study or interpret its results. As the links between the identified barriers and facilitators and existing frameworks and models of health behavior highlight, psychological theory may be highly beneficial to gain a better understanding of engagement with health apps and the design of more engaging apps [85]. It is therefore important to use theory in future studies about health app uptake and prolonged use as well as to use theory to inform app development.

### Limitations and Avenues for Future Research

Some concerns regarding the findings of the review arise from the included studies. Several of the included studies investigated reasons for health app (non)use more broadly. Although only studies were included that specifically addressed nutrition apps as a category of health apps, it could often not be determined whether an issue was raised in relation to nutrition apps or other categories of health apps. Thus, some barriers and facilitators might have been included in this review that did not refer to nutrition apps. Furthermore, most of the included studies did not provide information about anthropometrics or socioeconomic position of the participants, which makes it difficult to appraise generalizability of the findings. Although most studies reported the gender of the participants, females were overrepresented in many of the included studies, and 2 even focused exclusively on female participants [26,36]. While previous research suggests that nutrition app users are more likely to be female [17], including more male participants in future research would be desirable to address potential gender-specific needs (eg, aiming to lose weight vs aiming to gain muscle mass [86]), which might explain the lower adoption rates in males. Finally, selection bias, for example, due to publication bias, cannot be ruled out in both quantitative and qualitative studies [87]. It thus cannot be ruled out that relevant (unpublished) work may have been missed.

It is important to note that neither this review nor previous reviews on barriers and facilitators for nutrition app use can provide insights into the relative importance of the barriers and facilitators for the decision (not) to use a nutrition app. While the number of studies in which a reason was mentioned could be used as an indicator, it might also reflect research questions or questions and items used in the individual studies. Moreover, the grouping of barriers and facilitators into categories and clusters is somewhat arbitrary. Some categories in the framework might not be fully mutually exclusive, as for instance affinity for technology might also influence the perception of usability [88]. Notably, although Simblett et al [19] only identified 5 categories of barriers and facilitators for the use of remote tracking technologies, many of the underlying barriers and facilitators could also be mapped onto the framework presented in this review. Further research is thus needed to gain insight into interrelations of the identified barriers and facilitators and their grouping based on empirical data as well as to determine their relative importance (see, eg, [20] for wearables).

Furthermore, barriers and facilitators might differ between user groups, as has been highlighted in previous research on stage theories of behavior (eg, [64,89]). Differences between user groups could not be disentangled in this review as most studies reported barriers and facilitators for multiple user groups without indicating by which user group they were mentioned. One exception is the survey conducted by Murnane et al [29], which showed that current users experienced positive consequences of health app use such as feeling more healthy and energized, while apps were abandoned because they did not function properly or lacked desired features. Furthermore, the importance of barriers and facilitators might change while a nutrition app is used (see [90] for a discussion). As Baretta et al [91] showed in the context of apps for physical activity promotion, some features such as peer and coaching support might be more important for initial uptake, while, for example, proactive motivational features are more important for promoting continued use. Similarly, Sharpe et al [22] highlighted that usability might be more important for sustained engagement with nutrition apps. Future research should therefore explicitly compare different user groups and stages to provide valuable insights into how to promote uptake and continued use of nutrition apps by specifically targeting relevant barriers and facilitators.

Moreover, at least some of the barriers and facilitators identified in this review might not be specific to the use of nutrition apps, but related to changing eating behavior independent of the mode of delivery. For instance, from some of the included publications, it did not become clear whether a lack of interest in nutrition apps referred to the app itself or changing the behavior. More research is therefore needed to disentangle these effects.

### Conclusions

Through this systematic review, the literature on barriers to and facilitators for the uptake and continued use of nutrition apps was synthesized to provide a comprehensive overview of factors that hinder or promote use. A total of 328 barriers and facilitators were extracted from 28 publications and systematized in a framework with 23 subcategories clustered in 12 categories. Four higher-order clusters were formed that subsume barriers and facilitators related to technology, the individual, their interactions, and the social environment. Eight design guidelines were derived from the framework which app developers may implement to increase and prolong nutrition app use: enhance app usability, enhance food database usability, include effective BCT features, allow for personalization, anticipate positive and negative outcomes, advance trust in data accuracy, enhance data authority, and conserve smartphone resources. These design guidelines might be fruitful to support the aim of the European Union [92,93] to make web-based health promotion, including nutrition apps, more effective, user-friendly, and widely acceptable, and might ultimately contribute to achieving large-scale health effects.

## Appendix

Multimedia Appendix 1 PRISMA checklist for systematic reviews.

Multimedia Appendix 2 Identified categories per publication.

Multimedia Appendix 3 Quotes extracted from publications including assigned category.

Multimedia Appendix 4 Detailed overview of the included studies.

## Abbreviations

BCT: behavior change theory
DG: design guideline
