# Supplementary material for: Barriers to and Facilitators for Using Nutrition Apps: Systematic Review and Conceptual Framework
Source: JMIR Mhealth Uhealth. 2021 Jun 19;9(6):e20037. doi: 10.2196/20037 (PMC8409150; doi:10.2196/20037)
Supplement: Multimedia Appendix 2 [file mhealth_v9i6e20037_app2.docx]

**Multimedia Appendix 2:** Identified categories per publication.

|  |  |  |  |  |  |  |  |  |  |  |  |  |  |  |  |  |  |  |  |  |  |  |  |  |  |
| --- | --- | --- | --- | --- | --- | --- | --- | --- | --- | --- | --- | --- | --- | --- | --- | --- | --- | --- | --- | --- | --- | --- | --- | --- | --- |
| App user group |  | [C1A] Type of goal | [C1B] Goal attained | [C1C] Goal abandoned | [C2A] Lack of interest | [C2B] Declining motivation | [C3A] Daily routines | [C3B] Tracking habit | [C4] Lack of knowledge or skill | [C5A] Features linked to BCTs | C5B] Technical features | [C5C] Personalization | [C6A] Usability of the app | [C6B] Usability of the food tracking feature | [C7A] Data accuracy | [C7B] Data security and privacy | [C8] Technical issues | [C9] Financial costs | [C10A] Positive cognitive and emotional outcomes | [C10B] Positive behavioral and health outcomes | [C11A] Negative cognitive and emotional outcomes | [C11B] Negative behavioral and health outcomes | [C12A] Recommending use | [C12B] Social interactions | Categories identified within the publication |
| all | Aljuraiban [46] |  |  |  |  | x |  |  |  | x |  | x | x |  |  |  |  | x |  |  |  |  |  |  | 5 |
|  | Bhuyan et al. [41] | x |  |  |  |  |  |  |  |  |  |  |  |  |  |  |  |  |  |  |  |  |  |  | 1 |
|  | Chan et al. [45] |  |  |  | x |  |  |  | x | x |  | x | x |  |  |  |  |  |  |  |  |  | x | x | 7 |
|  | Dennison et al. [47] |  |  |  |  | x | x | x |  | x | x | x | x |  | x | x | x |  |  | x | x |  |  | x | 13 |
|  | Haithcox-Dennis et al. [30] | x |  |  | x |  |  |  | x |  |  |  | x |  |  |  |  | x |  | x |  |  | x |  | 7 |
|  | Jones et al. [25] | x |  |  |  |  |  |  |  |  |  |  | x |  |  |  |  |  | x | x |  |  |  |  | 4 |
|  | Krebs and Duncan [24] | x |  |  | x | x |  |  |  |  |  |  | x | x |  | x | x | x |  |  |  |  | x |  | 9 |
|  | Kwon et al. [54] |  |  |  |  |  |  |  |  |  |  |  |  |  |  |  |  |  |  | x |  |  | x | x | 3 |
|  | Murnane et al. [29] | x | x | x |  |  | x |  |  | x |  | x |  |  |  | x | x |  | x |  |  |  | x |  | 10 |
|  | Peng et al. [43] |  | x |  | x |  |  |  | x | x | x | x | x |  |  | x | x | x | x |  |  | x | x | x | 14 |
|  | Sarcona et al. [32] | x |  |  |  |  | x |  |  |  |  |  |  |  |  |  |  |  | x |  | x |  |  |  | 4 |
|  | Solbrig et al. [38] | x |  |  |  |  |  |  |  | x |  | x | x |  |  |  |  |  | x |  | x |  |  |  | 6 |
|  | Wang et al. [53] |  |  |  |  |  |  |  |  |  |  |  | x |  |  |  |  |  | x |  |  |  |  | x | 3 |
|  | Warnick et al. [52] |  |  |  |  |  |  |  |  |  |  |  | x |  |  |  |  |  |  |  |  | x |  |  | 2 |
|  | Zhou et al. [51] |  |  |  |  |  |  |  |  |  | x |  |  |  |  | x | x | x |  |  |  |  | x | x | 6 |
| users | Anderson et al. [50] |  |  |  |  |  |  |  |  | x | x | x | x |  |  | x |  |  | x | x |  |  |  |  | 7 |
|  | Choe et al. [39] | x |  |  |  |  |  |  |  | x |  |  | x | x |  |  |  |  |  |  |  |  |  |  | 4 |
|  | Flaherty et al. [36] | x |  |  |  | x |  |  |  | x | x | x | x |  | x | x |  |  | x |  |  |  |  |  | 9 |
|  | Gowin et al. [35] | x |  |  |  |  |  |  |  |  |  |  | x |  |  | x |  | x | x |  |  |  | x |  | 6 |
|  | Woldeyohannes and Ngwenyama [48] |  |  |  |  | x |  |  |  | x | x | x | x | x | x | x | x |  |  |  | x | x | x | x | 13 |
| users, ex-users | Cordeiro et al. [44] |  | x |  |  | x |  | x |  |  |  |  |  | x |  |  |  |  |  |  |  | x |  | x | 6 |
|  | Cordeiro, Epstein et al. [49] |  |  |  |  |  |  | x |  | x |  |  | x | x | x |  |  |  |  |  | x | x |  | x | 8 |
|  | Eikey and Reddy [26] | x |  |  |  |  |  |  |  |  |  |  |  |  |  |  |  |  |  |  |  | x |  |  | 2 |
|  | Lieffers et al. [33] | x | x |  |  | x | x |  | x | x | x | x | x | x | x |  | x | x |  |  | x | x |  | x | 16 |
|  | Oh and Lee [42] | x |  |  |  |  |  |  |  | x | x |  |  | x | x | x | x |  | x |  | x |  |  |  | 9 |
|  | Tang et al. [37] | x |  |  |  |  |  |  |  | x | x | x | x |  | x |  |  |  | x |  | x |  |  | x | 9 |
|  | West et al. [34] |  |  |  |  |  |  |  |  |  |  |  | x |  |  |  |  |  | x | x |  |  |  |  | 3 |
|  | Yuan et al. [40] | x |  |  |  |  |  | x |  |  |  |  |  |  |  |  |  | x | x |  |  |  |  |  | 4 |
|  | Number of publications in which a category was identified | 15 | 4 | 1 | 4 | 7 | 4 | 4 | 4 | 14 | 9 | 11 | 19 | 7 | 7 | 10 | 8 | 8 | 13 | 6 | 8 | 7 | 9 | 11 |  |
