# Supplementary material for: Barriers to and Facilitators for Using Nutrition Apps: Systematic Review and Conceptual Framework
Source: JMIR Mhealth Uhealth. 2021 Jun 19;9(6):e20037. doi: 10.2196/20037 (PMC8409150; doi:10.2196/20037)
Supplement: Multimedia Appendix 4 [file mhealth_v9i6e20037_app4.docx]

**Multimedia Appendix 4:** Detailed overview of the included studies.

| **Publication** | **N** | **Target group** | **App user group** | **Study design** | **Type of apps** | **Location** | **Theory?** | **Results clustered for user groups?** | **Results clustered for barriers/ facilitators?** | **Setting** |
| --- | --- | --- | --- | --- | --- | --- | --- | --- | --- | --- |
|  |  |  |  |  |  |  |  |  |  |  |
| Aljuraiban [46] | 1191 | adults | all | quantitative | health apps | Saudi Arabia | - | Partially (reasons for discontinuation based on ex-users) | yes | Real world |
| Anderson, et al. [50] | 22 | adults | user | qualitative | health apps | Australia | Technology Acceptance Model, Health Information Technology Acceptance Model | no | no | Real world |
| Bhuyan et al. [41] | 3677 | adults | all | quantitative | health apps | US | - | Partially (results on use characteristics only based on users) | no | Real world |
| Chan et al. [45] | 20 | adolescents (13 - 18 years of age) | all | qualitative | health apps | US | - | no | yes | Real world |
| Choe et al. [39] | 52 | adults | user | qualitative | health apps | international (US, UK, Singapore) | - | no | no | Real world |
| Cordeiro, Epstein et al. [49], Study 1 | 141 | adults | user, ex-user | quantified | nutrition apps | N/A | - | no | Focus on barriers | Real world |
| Cordeiro, Epstein et al. [49], Study 2 | 5526 posts | N/A | user, ex-user | quantified | nutrition apps | N/A | - | no | Focus on barriers | Real world |
| Cordeiro, Bales et al. [44] | 257 | adults | user, ex-user | quantified | nutrition apps | N/A | - | no | Focus on barriers | Real world |
| Dennison et al. [47] | 19 | adults | all | qualitative | health apps | UK | - | no | no | Real world |
| Eikey & Reddy [26] | 16 | adults | user, ex-user | qualitative | nutrition apps | US | - | no | Yes (but focus on negative outcomes) | Real world |
| Flaherty et al. [36] | 12 | adults | user | qualitative | nutrition apps | Ireland | Consumer engagement theory | no | no | Field study (were encouraged to use specific apps as naturally as possible) |
| Gowin et al. [35] | 27 | adults | user | qualitative | health apps | US | - | no | no | Real world |
| Haithcox-Dennis et al. [30] | 1487 | adults | all | quantitative | health apps | US | - | no | no | Real world |
| Jones et al. [25] | 195 | adults | all | quantitative | health apps | US | - | Partially (for results on nutrition app use satisfaction only current users were included) | Only Facilitators | Real world |
| Krebs & Duncan [24] | 1604 | adults | all | quantitative | health apps | US | - | yes | yes | Real world |
| Kwon et al. [54] | 391 | adults | all | quantitative | health apps | US | Theory of Reasoned Action, Theory of Planned Behavior, TAM, Social Cognitive Theory | no | no | Real world |
| Lieffers et al. [33] | 24 | adults | user, ex-user | qualitative | nutrition apps | Canada | Diffusions of Innovations Framework, TAM | no | no | Real world |
| Murnane et al. [29] | M = 1839 responses per question | adults | all | quantitative | health apps | US | - | Partially (reasons for discontinuation based on ex-users) | yes | Real world |
| Oh & Lee [42] | N/A | N/A | user, ex-user | quantified | health apps | N/A | - | no | no | Real world |
| Peng et al. [43] | 44 | adults | all | qualitative | health apps | US | - | yes | yes | Real world |
| Sarcona et al. [32] | 401 | adults | all | quantitative | health apps | US | - | Partially (results on use characteristics only based on users) | yes | Real world |
| Solbrig et al. [38] | 24 | adults | all | qualitative | nutrition apps | UK | Elaborated Intrusion Theory | no | yes | Real world |
| Tang et al. [37] | 19 | adults | user, ex-user | qualitative | nutrition apps | UK | - | no | Focus on Facilitators | Real world |
| Wang et al. [53], Study 1 | 23 | adults | all | qualitative | health apps | Norway | Theory of planned behavior | no | no | Real world |
| Wang et al. [53], Study 2 | 500 | adults | all | quantitative | health apps | Norway | Theory of planned behavior | no | no | Real world |
| Warnick et al. [52] | 18 | adults | all | qualitative | health apps | US | Integrated behavioural model | no | yes | Real world |
| West et al. [34] | 217 | adults | user, ex-user | quantitative | nutrition apps | US | Health belief model, Theory of Planned Behavior, Social Cognitive theory, | no | Focus on Facilitators | Real world |
| Woldeyohannes & Ngwenyama [48] | 11 | adults | user | qualitative | health apps | Canada | UTAUT 2 | no | no | Real world |
| Yuan et al. [40] | 317 | adults | user, ex-user | quantitative | health apps | US | UTAUT 2 | no | no | Real world |
| Zhou et al. [51] | 117 | adults | all | mixed | health apps | US | - | no | yes | Real world |
